# Coupling of electrochemically triggered thermal and mechanical effects to aggravate failure in a layered cathode

**DOI:** 10.1038/s41467-018-04862-w

**Published:** 2018-06-22

**Authors:** Pengfei Yan, Jianming Zheng, Tianwu Chen, Langli Luo, Yuyuan Jiang, Kuan Wang, Manling Sui, Ji-Guang Zhang, Sulin Zhang, Chongmin Wang

**Affiliations:** 1 Environmental Molecular Sciences Laboratory, Pacific Northwest National Laboratory, 902 Battelle Boulevard, Richland, WA 99352 USA; 2 Institute of Microstructure and Properties of Advanced Materials, Beijing University of Technology, No. 100, Pingleyuan, Chaoyang District, Beijing, 100124 PR China; 3 Energy and Environment Directorate, Pacific Northwest National Laboratory, 902 Battelle Boulevard, Richland, WA 99352 USA; 4 Department of Engineering Science and Mechanics, Pennsylvania State University, University Park, PA 16802 USA

## Abstract

Electrochemically driven functioning of a battery inevitably induces thermal and mechanical effects, which in turn couple with the electrochemical effect and collectively govern the performance of the battery. However, such a coupling effect, whether favorable or detrimental, has never been explicitly elucidated. Here we use in situ transmission electron microscopy to demonstrate such a coupling effect. We discover that thermally perturbating delithiated LiNi_0.6_Mn_0.2_Co_0.2_O_2_ will trigger explosive nucleation and propagation of intragranular cracks in the lattice, providing us a unique opportunity to directly visualize the cracking mechanism and dynamics. We reveal that thermal stress associated with electrochemically induced phase inhomogeneity and internal pressure resulting from oxygen release are the primary driving forces for intragranular cracking that resembles a “popcorn” fracture mechanism. The present work reveals that, for battery performance, the intricate coupling of electrochemical, thermal, and mechanical effects will surpass the superposition of individual effects.

## Abstract (web summary)

Electrochemical processes induce thermo-mechanical effects that mediate battery performance. Here the authors directly visualize cracking dynamics in a thermally perturbed, delithiated LiNi_0.6_Mn_0.2_Co_0.2_O_2_ cathode to demonstrate coupling between thermal, mechanical and electrochemical factors.

## Introduction

Since rechargeable lithium-ion batteries (LIB) were first commercialized in the 1990s, continuous efforts have focused on developing high-energy-density LIBs, which can be accomplished by simultaneously increasing the energy densities of both the cathode and the anode^1–3^. Silicon-based nanostructured composites and lithium metals are ideal candidates for anodes^4–6^. For cathodes, layered lithium transition metal oxides have been considered as a class of major players for next-generation LIBs^7,8^. Specifically, nickel (Ni)-rich-layered cathodes are attracting growing attentions because of their high discharge capacities (~200–220 mA h g^–1^), high rate capability, and reduced cost^9–11^. To maximize lithium-source utilization, cathodes often are charged (delithiated) to a high voltage. However, excessive extraction of lithium from layered cathodes could cause structural instability, along with other parasitic degradation pathways in LIB cells. It has been observed that high voltage cycling can lead to rapid performance decay, which may be attributed to aggravating redox reactions at the cathode–electrolyte interface^12^, cathode surface-phase transformation^13^, active material dissolution into the electrolyte^14^, electrolyte decomposition^12^, cathode passivation layer formation^15^, intergranular cracking^16–18^, and intragranular cracking^19,20^. The origins of these detrimental factors has been correlated to electrochemical, thermal, and mechanical effects^21^. In principle, the electrochemical process often can trigger thermal and mechanical processes, and in turn, the thermo-mechanical effects can mediate the electrochemical process. Thus, these three processes are intimately coupled in ways that result in mutual reinforcement; that is, together they often result in an aggravate effect on battery performance. In the past, much work has focused on delineating individual effects, and the coupling effect has not been explored.

In this work, we integrate in situ transmission electron microscopy (TEM), in situ X-ray diffraction (XRD), and modeling to demonstrate such a coupling effect on the intragranular cracking of a Ni-rich LiNi_0.6_Mn_0.2_Co_0.2_O_2_ (NMC622) layered cathode. We show that in situ heating of delithiated NMC622 particles, even at initial delithiation from the pristine state, leads to explosive nucleation and propagation of intragranular cracks. Such a phenomenon is absent in fully discharged particles (with lithium being reinserted). Combining ex situ and in situ TEM observations with chemo-mechanical modeling, we reveal that, in response to the temperature increase, electrochemically induced phase inhomogeneity and oxygen evolution cause thermal stress and internal pressure, respectively, which constitute the two primary driving forces for intragranular cracking. The present work demonstrates that intimate coupling of the electrochemical, thermal, and mechanical processes leads to much more severe cathode degradation.

## Results

### Electrochemical properties and cycling-induced defect configurations

**Fig. 1:**
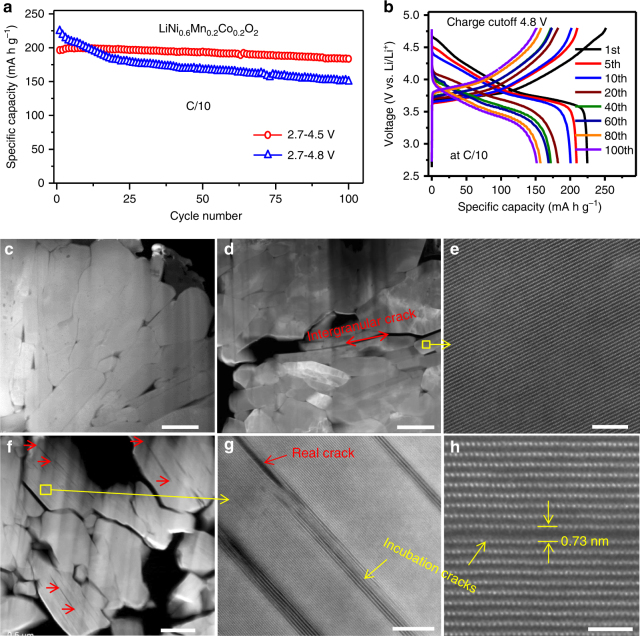
Cycling performance and cracks of LiNi_0.6_Mn_0.2_Co_0.2_O_2_. **a** Capacity decay and **b** corresponding charge–discharge voltage profile evolution at cycling window 2.7–4.8 V. Cross-sectional high angle dark field (HAADF) images from (**c**) pristine sample **d**, **e** after 100 cycles at 2.7–4.5 V, **f**–**h** after 100 cycles at 2.7–4.8 V. Red arrow in (**d**) highlights intergranular cracks formed during cycling. Red arrows in (**f**, **g**) highlight some intragranular cracks within primary particles. Yellow arrows in (**g**, **h**) highlight incubation cracks. The scale bars are 500 nm in (**c**–**f**), 10 nm in (**g**) and 2 nm in (**h**)

Li/NMC622 half cells were tested at a C/10 rate at room temperature in 1-M LiPF_6_ EC/EMC electrolyte. Two cycling windows were used for the half cells, 2.7–4.5 and 2.7–4.8 V. Figure 1a displays plots of discharge capacities at different cycle numbers, which clearly shows that the cell cycled in the 2.7–4.8 V voltage window exhibits much faster capacity decay, especially during the first 20 cycles. After 20 cycles, the capacity decay slowed but was still faster than the cell cycled at 2.7–4.5 V voltage window. After 100 cycles, the cell cycled in the 2.7–4.5 V voltage window showed 90% capacity retention while the cell cycled in the 2.7–4.8 V voltage window only had 66% retention. High charge cutoff voltage not only resulted in faster capacity decay, but also caused serious voltage fading, as shown in Fig. 1b. In the first cycle, charging NMC622 to 4.8 V obtained a specific charge capacity 252 mA h g^−1^, indicating 91% of the lithium ions were extracted from the cathode lattice. When charged to 4.5 V, a specific charge capacity of 223 mA h g^–1^ was achieved, accounting for 81% of the theoretical capacity (276 mA h g^–1^). Although charging to high voltages can indeed maximize lithium utilization in the first several cycles, rapid capacity and voltage fading leads to relatively poor cyclability. Using focused ion beam (FIB) and scanning electron microscopy (SEM), we prepared cross-sectional TEM specimens for a detailed investigation. As shown in Supplementary Fig.1, the cross-sectional SEM images reveal the formation of intergranular cracks in the layered electrodes cycled at 4.5 and 4.8 V. In contrast, intragranular cracks were observed only in the electrode cycled at 4.8 V. As shown in Fig. 1c–h, we compared cross-sectional scanning TEM of high angle annular dark field (STEM-HAADF) images of the pristine electrode and those after 100 cycles with high charge cutoff voltages at 4.5 and 4.8 V, respectively. All intragranular cracks follow a strict crystallographic preference along the (003) layered plane. These features are consistent with findings from our previous study on LiNi_1/3_Mn_1/3_Co_1/3_O_2_ (NMC333) particles^19^ and confirm high voltage cycling as the key factor that causes intragranular cracking.

### Critical voltage for intragranular cracking

To precisely determine the critical high cutoff voltage for intragranular cracking, we charged the NMC622 to 4.55, 4.6, and 4.65 V. As shown in Supplementary Fig. 2, XRD data show that the (003) plane expands due to charging, and intragranular cracks are observed in the sample charged to 4.65 V. Below 4.65 V, intragranular cracks were not observed. Therefore, for NMC622, we determined that the critical voltage for intragranular cracking lies in the range of 4.60–4.65 V (as shown in Supplementary Fig. 2b). Our results consistently show that high voltage cycling results in deep delithiation, higher internal strain, and even phase transformation^22^.

**Fig. 2:**
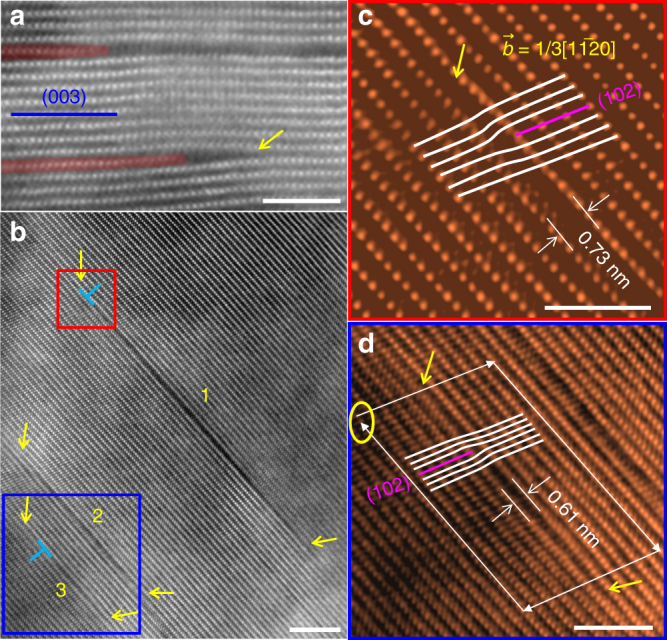
Edge dislocations as nucleation sites of intragranular cracks in cycled LiNi_0.6_Mn_0.2_Co_0.2_O_2_. **a** Cracks connecting with edge dislocations with extra (003) planes. **b** Three intragranular cracks labeled as “1”, “2” and “3”. The yellow arrows indicate crack ends. **c** and **d** the enlarged region marked by the red and blue squares in (**b**). (102) extra planes are identified in “1” and “3” as highlighted in (**c**) and (**d**), respectively, verifying the edge dislocation in each crack. The scale bars are 3 nm in (**a**), (**c**) and (**d**); 5 nm in (**b**)

Intergranular cracking, frequently described as mechanical failure, is considered to be a major contributor to performance decay of layered cathodes^16,18,23^. because they create new cathode–electrolyte interfaces leading to unwanted surface degradation, electrolyte consumption, and poor electronic conductivity. Intragranular cracks, although it is hard to quantify their contribution to the overall performance decay, they will ultimately evolve into large cracks, acting like intergranular cracks to negatively impact cycling performance. Furthermore, the density of intragranular cracks are orders of magnitude higher than that of intergranular cracks, leading to destruction of the integrity of layered cathodes. Therefore, understanding the nucleation and propagation of intragranular cracks is of great importance for optimizing layered cathodes. In Fig. 2, we show that edge dislocations are important nucleation sites of intragranular cracks. Figure 2a shows an edge dislocation with a (003) extra plane that can initiate intragranular cracks^19^. Fig. 2b–d shows that other edge dislocations with (102) extra plane also can initiate intragranular cracks. Compared with an edge dislocation of (003) extra plane, an edge dislocation of (102) extra plane can glide on the (003) plane. It is likely that this gliding of edge dislocation on the (003) plane can promote crack propagation. It should be noted that when viewing an edge dislocation along its Burger’s vector, the extra plane cannot be seen (see Supplementary Fig. 3).

### Direct in situ TEM observation of intragranular cracking dynamics

**Fig. 3:**
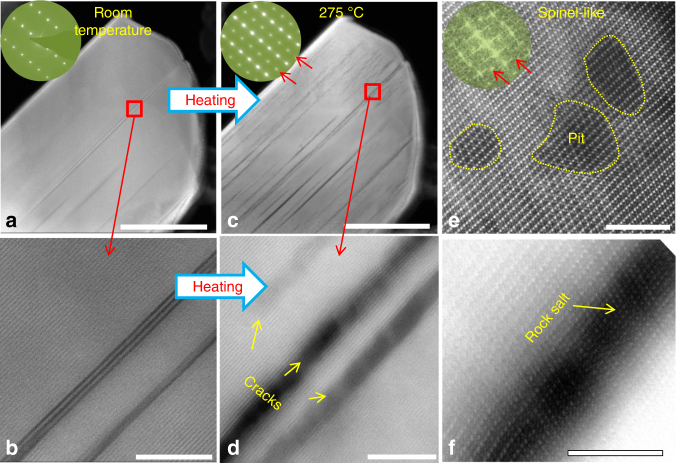
In situ heating-induced crack nucleation and propagation. The LiNi_0.6_Mn_0.2_Co_0.2_O_2_ (NMC622) was delithiated by charging to 4.7 V vs. Li metal. **a** and **b** High angle annular dark field (HAADF) images from delithiated NMC622 before heating (room temperature). **c**–**f** HAADF images after heating to 275 °C. Selected area electron diffraction (SAED) patterns and fast Fourier transformation images in (**a**), (**c**), and (**e**) show the overall lattice change during heating. **d**, **e**, and **f** show local lattice structure change at crack regions and crack-free regions. The scale bars are 200 nm in (**a**) and (**c**); 10 nm in (**b**) and (**d**); and 4 nm in (**e**) and (**f**)

Although the postmortem STEM analysis captures the general features of the intragranular cracks, the cracking dynamics are critically missing. In situ STEM observations provide an opportunity to directly monitor the nucleation and propagation of intragranular crack by heat treatment of the delithiated NMC622 samples. This approach mimics the thermal effect of the electrochemical process that involves exothermic reactions. The delithiated NMC622 samples were prepared by charging the cathode electrode to 4.7 V in a half-cell configuration of Li metal–NMC622. Prior to in situ heating, the delithiated NMC622 particles were examined using STEM-HAADF imaging. It is interesting to note that even the initial charging (delithiated from the pristine state) can lead to formation of intragranular cracks, as shown in Fig. 3a, b and Supplementary Figs. 4, 5a, c, and 6a. Despite cracking, the layered structure of the material was preserved, as confirmed by lattice images and selective area electron diffraction (SAED) patterns (Fig. 3a, b and Supplementary Fig. 4). Intriguingly, when the delithiated samples were heated to high temperatures, many new cracks were explosively introduced and the pre-existing cracks propagated. In Fig. 3, the particle starts generating new cracks at 275 °C. High-resolution STEM-HAADF imaging revealed that a rock-salt phase formed at the free surface of the cracks (Fig. 3f), thereby widening the crack. In the crack-free regions, the lattice showed spinel features, as evidenced by the fast Fourier transformation inset in Fig. 3e, which was consistent with the SAED pattern shown in the inset of Fig. 3c. Consistent with a previous report^24^, pit formation also was observed (see Fig. 3e), but it occurred at a lower temperature in our study. More examples of heating-induced cracking are provided in Supplementary Fig. 5. Cracking dynamics also are shown in Supplementary Movie 1 and snapshots in Supplementary Fig. 6, where cracks nucleated from both the surface and bulk upon heating. It should be noted that due to intragranular cracking along the (003) lattice planes, for the in situ STEM observation of the nucleation and propagation of the intragranular cracking, it is essential to tilt the crystal to the orientation such that the (003) plane is parallel with the electron beam, otherwise such a cracking event will not be visible as shown in Supplementary Fig. 7.

**Fig. 4:**
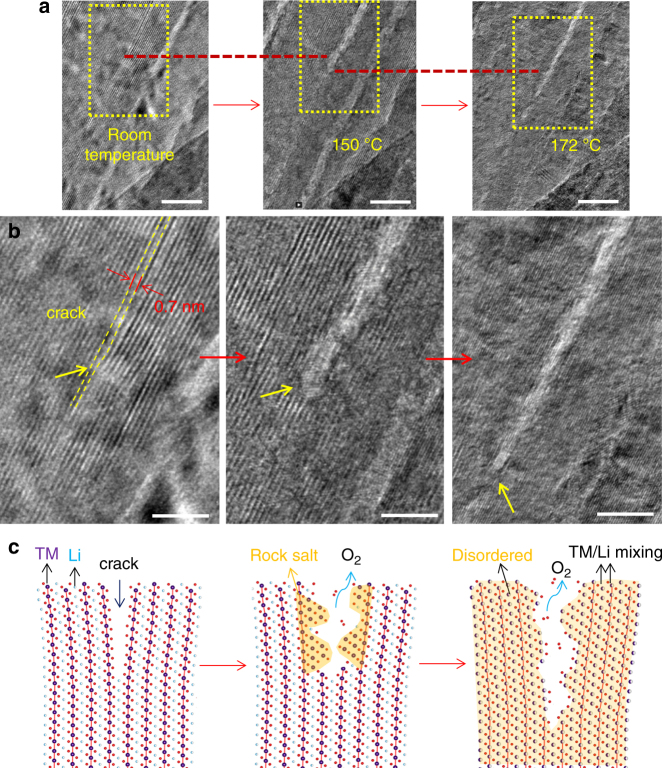
In situ observing crack propagation with high-resolution transmission electron microscopy. **a** Low magnification image series showing crack propagation during heating, where the dash-red lines mark the same location. **b** Lattice images taken from the regions marked by the dash-yellow rectangular in (**a**), showing the structure change, crack widening, and propagation during heating, where the yellow arrows indicate the crack tip. **c** Schematic illustration of the cracking process during heating. The red arrows indicate the progression of heating. The scale bars are 10 nm in (**a**) and 5 nm in (**b**). “TM” in (**c**) is the abbreviation of “transition metal”

To observe the initiation of crack propagation and reveal the underlying atomistic mechanism, we conducted in situ high-resolution transmission electron microscopy (HRTEM) observations on a crack tip to directly monitor lattice evolution during heating. As shown in Fig. 4a, when the temperature reached 150 °C, the crack contrast became brighter and the crack gap widened. HRTEM imaging confirmed that the crack center was transformed into the rock-salt phase, while the layered structure remained in crack-free regions. The phase transformation indicates poor thermal stability at the crack surface and heating-induced thermal decomposition preferentially occurs at cracked regions. From room temperature to 150 °C, the crack propagated a few nanometers. Heating the sample to 172 °C resulted in further crack propagation, as shown in Fig. 4a, b. Above 172 °C, the crack tip arrested, suggesting that the thermally induced driving force for crack propagation no longer exceeds the fracture resistance. Based on the in situ HRTEM observations, we schematically illustrate in Fig. 4c the crack-propagation process during heating. As the first step, the crack surface region is locally decomposed into the rock-salt structure, accompanied with release of oxygen. As the temperature is increased further, sufficiently high thermal stress is generated in the sample, driving crack propagation. The newly generated crack surfaces undergo thermal decomposition and release oxygen. Crack-free regions concurrently undergo thermal decomposition, causing disordering of the layered lattice.

**Fig. 5:**
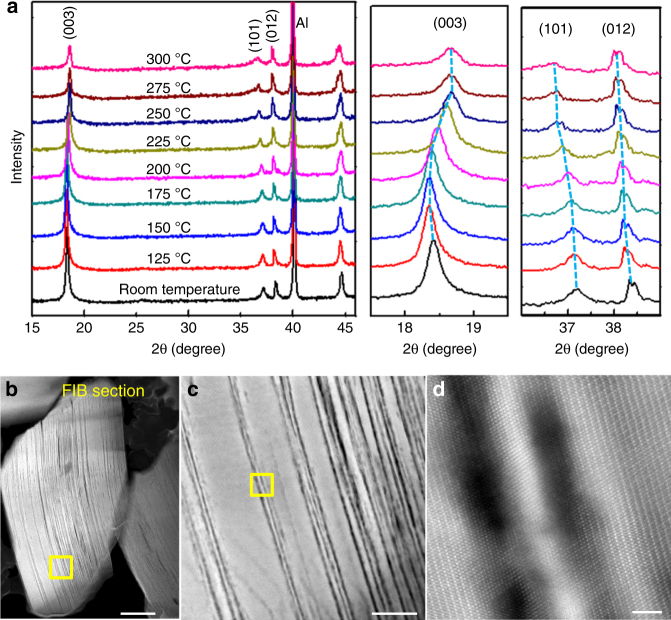
Evolution of LiNi_0.6_Mn_0.2_Co_0.2_O_2_ charged to 4.7 V during heating. **a** In situ X-ray diffraction (XRD) measurements. **b**–**d** Direct high angle annular dark field (HAADF) observation of the inner bulk degradation at gradually increased magnifications, enabled by focus ion beam (FIB) milled cross-section sample. The yellow square region in (**b**) is enlarged as (**c**) and the yellow square region in (**c**) is enlarged as (**d**). The scale bars are 200 nm in (**b**), 50 nm in (**c**), and 2 nm in (**d**)

To further validate the in situ TEM observations, we conducted in situ XRD measurements on the delithiated NMC622 samples. As shown in Fig. 5a, the (003) peak shifts to a high angle, and its peak intensity significantly decreases due to heating. Correspondingly, the (101) peak shifts to a low angle, indicating larger in-plane lattice parameters (*a*, *b*). The XRD signals are taken from bulk materials, thus providing compelling evidence of an overall structural change of the delithiated NMC622. Supplementary Fig. 8 shows the intragranular cracking and thermal decomposition that occurred in the temperature range 210–230 °C. Furthermore, we prepared a cross-section specimen using FIB to directly observe the interior of the bulk after heating to 230 °C. As shown in Fig. 4b–d and Supplementary Fig. 9, cracking and thermal decomposition take place at the interior of the particles, suggesting that the phenomenon is a bulk degradation process rather than a surface-mediated behavior.

While intragranular cracking nucleation and propagation observed in our experiments resulted from external heating, we believe the driving force in heating-induced cracking is similar to cracking induced by electrochemical cycling, which often involves exothermic reactions that release both gas molecules and heat. Heat accumulated during electrochemical cycling could lead to a significant temperature increase during battery operation and eventually thermal runaway. Thus, heating-accelerated structural degradation has been accepted as a validated approach for the study of layered cathode using XRD^25–27^ and TEM^8,24,28^. Supplementary Fig. 10 shows that fully discharged NMC622 particles (after 100 cycles in a voltage window of 2.7–4.5 V) do not show any intragranular cracking even when heated to 600 °C; only surface layer phase transitions are observed. Such distinctly different responses of the crystals to heating between the delithiated state and the fully discharged (with Li re-inserted) state (Supplementary Fig. 10) clearly demonstrate the coupled effect among the electrochemical, thermal, and mechanical processes.

At low magnification, cycling-induced cracks appear to be similar to heating-induced cracks. However, detailed observations at high magnification reveals that cycling-induced cracks result from the cleaving of neighboring (003) planes, whereas heating-induced cracks contain rock-salt phases on the crack surfaces (Fig. 3b, d). The rock-salt structure arises from decomposition of the original layered structures. Thermal decomposition also leads to a spinel-feature lattice at the subsurfaces and pit formation in the crack-free region. According to previous studies^24,29^, delithiated layered structures are transformed into a spinel structure and/or a rock-salt structure, depending on the compositions and heating temperatures. In our case, heating-induced thermal decompositions are:

1 $${\rm Li}_{1 - x}{\mathrm{M}}{\rm O}_2 \to (1 - x) \cdot {\rm Li}\mathrm{M}_2{\rm O}_4({\rm spinel}) \\ + (2x - 1) \cdot {\mathrm{M}}{\rm O}({\rm rock\,salt}) + (2x - 1)/2 \cdot {\rm O}_2({\rm gas})$$

2 $${\rm Li}_{1 - x}{\mathrm{M}}{\rm O}_2 \to (1 - x)/2 \cdot {\rm Li}_2\mathrm{O} \\ + {\mathrm{M}}{\rm O}({\rm rock\,salt}) + (1 + x)/2 \cdot {\rm O}_2({\rm gas})$$

where M *=* Mn, Co, and Ni, and *x* is the extracted lithium (0 < *x* *<* 1). At the crack-free region, thermal decomposition is mild and forms the spinel-like lattice. At the crack surface, decomposition is severe and leads to formation of the rock-salt phase. According to Eqs. (1) and (2), we can learn that decomposition of a layered cathode requires a release of oxygen. Therefore, it is kinetically easier for oxygen to be released from the crack surface than from the inner bulk, which explains the preferential decomposition of crack surface layers. Lithia leads to weak contrast under STEM-HAADF imaging, showing pit characteristics. Based on SAED and electron energy loss spectroscopy (EELS) analysis, as shown in Supplementary Fig. 11, lithia is in an amorphous state and enriched in the dark region (pit position). According to Eqs. (1) and (2), thermal decomposition results in phase transformation and also oxygen release. Oxygen release also indicates the exothermic nature of the reactions, which may lead to thermal runaway and safety issues for the battery cells. To further elucidate cracking mechanisms in NMC622 particles during heating, we note that thermally induced material decomposition and cracking are two highly coupled processes. First, the crack surface facilitates thermal decomposition, leading to the formation of rock-salt phase at crack surfaces and spinel phase in the subsurface. This process transforms the original layered structure to a heterogeneous material. Moreover, the starting particle may not be fully delithiated, giving rise to inhomogeneous lithium distribution that also contributes to the material heterogeneity. Because of the different thermal expansion coefficients of the heterogeneous phases, heating induces incompatible strain and hence significant thermal stress, a critical mechanism that drives crack nucleation and propagation. In addition, if the cracks do not fully extend to the outer surface of the particles, the oxygen released during thermal decomposition may be trapped within the cracks. At elevated temperatures, the trapped oxygen molecules exert internal pressure onto the incubation and the real crack surfaces, presenting an additional driving force for crack nucleation and propagation. Furthermore, our EELS analysis suggests that cracking is accompanied by oxygen evolution. As shown in Supplementary Fig. 12, the pre-peak on the oxygen K-edge is depressed, indicating the formation of oxygen vacancies^30^. Therefore, the oxygen concentration in the crack region is low. Thus, intergranular cracking and material decomposition mutually enhance one another, presenting a fracture mechanism that resembles “popcorn” cracking under heating. It should be noted that crack growth will relax the local strain, and to a certain point, the driving force cannot sustain further cracking. That explains why the crack density reaches a maximum during heating. Once the internal strain is fully relaxed, even heating the sample to higher temperatures will not lead to further crack growth or creation.

## Discussion

**Fig. 6:**
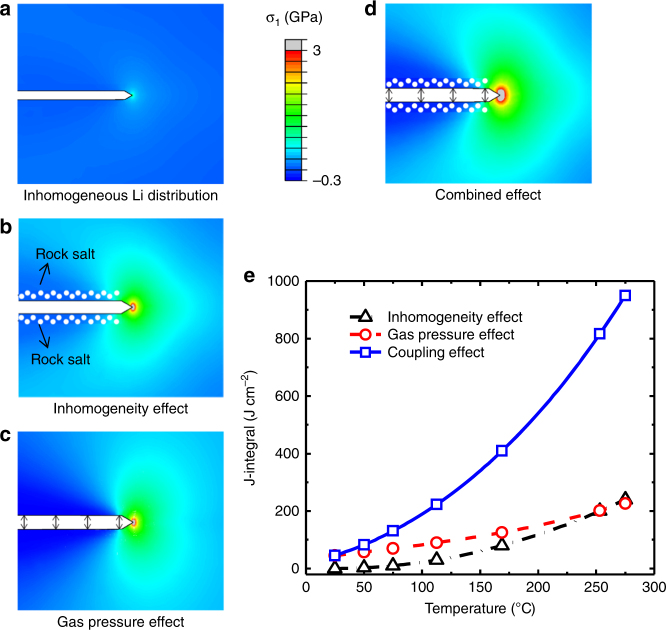
Modeling of the driving force for crack propagation in heated LiNi_0.6_Mn_0.2_Co_0.2_O_2_. The driving force is indicated by stress at the crack tip and measured by *J*-integral, upon considering material heterogeneity and crack surface pressure by the released oxygen gas. **a** Driving force for crack propagation without heat treatment considering inhomogeneous lithium distribution (*J* = 45 J cm^‒2^). **b** Driving force for crack propagation with heat treatment (heated to 275 °C) considering the material inhomogeneity, in terms of both inhomogeneous lithium concentration and rock-salt phase formation (*J* = 226 J cm^‒2^). **c** Driving force for crack propagation considering the surface pressure exerted by thermal decomposition generated oxygen molecules trapped within the crack at elevated temperature of 275 °C (*J* = 240 J cm^−2^). **d** Fracture driving force when considering both the effects in (**b**) and (**c**) (*J* = 950 J cm^‒2^). **e** *J*-integrals of the crack as a function of temperature

To elucidate the coupling effect, we performed comprehensive mechanistic analyses on different origins of driving forces for crack propagation in NMC622 particles (see Supplementary Methods and Supplementary Table 1). In Fig. 6a, we first consider the driving force due to inhomogeneous lithium distribution without heat treatment, assuming the lithium concentration linearly increases from the outer surfaces (almost fully delithiated) to the center (~20% lithium remains). This inhomogeneous distribution causes mismatched chemical strain, leading to stress concentration near the crack tip. The driving force for crack propagation measured by the *J*-integral is relatively small (*J* *=* 45 J cm^−2^). In Fig. 6b, we consider material inhomogeneity stemming from both phase transformation and inhomogeneous lithium concentration. In particular, we set the crack surface as the rock-salt phase with a different thermal expansion coefficient than the rest of the domain; that is, isotropic expansion in the rock-salt phase and anisotropic expansion in the layered-structure phase. The calculated thermal stress (heated to 275 °C) in the material is substantial, and the *J*-integral also is relatively high (*J* *=* 226 J cm^–2^). Note that in an otherwise homogeneous material the thermal stress should vanish and so as the *J*-integral. In Fig. 6c, we assume that the material is homogeneous, but consider the crack surface pressure due to the presence of the released oxygen molecules using the van der Waals equation at elevated temperature (275 °C). Our simulations show that the gas pressure also generates a considerable driving force for crack propagation (*J* *=* 240 J cm^–2^). In Fig. 6d, we combine all the effects, which leads to a very high driving force (*J*-integral, *J* *=* 950 J cm^–2^). By setting the extent of thermal decompositions linearly related to temperature during heat treatment, Fig. 6e shows that the driving force is strongly dependent on the temperature in the NMC622 particles. Our results indicate that the fracture driving force at elevated temperatures may well exceed the fracture resistance of the material due to the combined effect, leading to the crack propagation observed in the experiments.

To understand why crack nucleation and propagation is less prevalent in thin samples, one notes that thin samples tend to be more homogeneous. This is because heating-induced phase transformation may take place everywhere due to the large surface-to-volume ratio of the thin samples. Thus, the thermal stress induced by material inhomogeneity is negligible in thin samples. In addition, it is unlikely that thin samples are able to trap oxygen molecules within the cracks, as the cracks can easily extend to the outer surface of the thin samples. Thus, both mechanisms for crack propagation prevalent in thick samples are weaker or absent in thin samples. This explains why crack nucleation and propagation are rarely seen in thin samples, while phase transformation is the dominant phenomenon observed in the experiments (Fig. 4).

In situ STEM observations reveal that the response of delithiated NMC622 to heat treatment resembles the electrochemically cycling-induced battery performance decay, featuring nucleation, and propagation of intragranular cracks. Characteristically, heating-induced intragranular cracking is accompanied with surface phase transformation and oxygen release. Comprehensive mechanistic analyses and modeling reveal that thermal stresses resulting from material phase heterogeneity and internal pressure associated with oxygen release constitute two major driving forces for intergranular cracking. Taken together, the observed intragranular cracking resembles a popcorn fracture mechanism under heating. Our studies offer insights into how the electrochemical process triggers thermal and mechanical processes in battery operation, and how these effects are intimately coupled and mutually strengthened in the degradation of layered cathode materials. This fundamental understanding also sheds light on mitigation of battery-material degradation under complicated electrochemical and thermo-mechanical conditions.

## Methods

### Cathode material and cell test

NMC622 pristine electrode laminates were provided by the Cell Analysis, Modelling, and Prototyping Facility at Argonne National Laboratory (pristine powders are commercially available and are manufactured by TODA KOGYO Company, Japan). All the cathode electrodes were cycled at a *C*/10 rate (1*C* = 180 mA g^–1^) in voltage ranges of 2.7–4.5, 2.7–4.7, and 2.7–4.8 V (details can be found in Ref. ^19^).

### Microstructure characterizations

FIB/SEM imaging and TEM specimen preparation by FIB lift out were conducted on a FEI Helios DualBeam FIB operating at 1–30 kV. STEM observation was conducted on FEI Titan 80-300 TEM with probe corrector. In STEM mode the beam current is 60 pA. For high-resolution lattice imaging, the beam dose is around 10^6^ e Å^–2^. HRTEM observations were conducted on FEI Titan 80-300 Environmental TEM with objective lens corrector. The beam current is 140 pA. The in situ TEM heating holder is carried using a double tilt holder based on resistant heating coil made by Gatan (Gatan Inc., Pleasanton, CA, USA). The sample was heated at a constant rate of 5 °C min^–1^ from room temperature and the temperature is measured using a thermal couple integrated into the holder. In situ XRD experiments were carried out using a Bruker D8 advace X-ray diffractometer with a heating stage.

### Data availability

Data that support the plots in this paper and other findings of this study are available from the corresponding authors upon reasonable request.

## Electronic supplementary material

Supplementary Information

Description of Additional Supplementary Files

Supplementary Movie 1

## References

[1] Thackeray MM (2007). Li_2_MnO_3_-stabilized LiMO_2_ (M = Mn, Ni, Co) electrodes for lithium-ion batteries. J. Mater. Chem..

[2] Zheng J (2012). Enhanced Li^+^ ion transport in LiNi_0.5_Mn_1.5_O_4_ through control of site disorder. Phys. Chem. Chem. Phys..

[3] Sun YK (2012). Nanostructured high-energy cathode materials for advanced lithium batteries. Nat. Mater..

[4] Lu J (2016). The role of nanotechnology in the development of battery materials for electric vehicles. Nat. Nanotechnol..

[5] Kim H, Lee EJ, Sun YK (2014). Recent advances in the Si-based nanocomposite materials as high capacity anode materials for lithium ion batteries. Mater. Today.

[6] Lin D, Liu Y, Cui Y (2017). Reviving the lithium metal anode for high-energy batteries. Nat. Nanotechnol..

[7] Li W, Song B, Manthiram A (2017). High-voltage positive electrode materials for lithium-ion batteries. Chem. Soc. Rev..

[8] Rozier P, Tarascon JM (2015). Review—Li-rich layered oxide cathodes for next-generation Li-ion batteries: chances and challenges. J. Electrochem. Soc..

[9] Luo L (2015). Surface-coating regulated lithiation kinetics and degradation in silicon nanowires for lithium ion battery. ACS Nano.

[10] Yoon CS, Choi MH, Lim BB, Lee EJ, Sun YK (2015). Review—high-capacity Li[Ni_1-x_Co_x/2_Mn_x/2_]O_2_ (x=0.1, 0.05, 0) cathodes for next-generation Li-ion battery. J. Electrochem. Soc..

[11] Manthiram A, Knight JC, Myung ST, Oh SM, Sun YK (2016). Nickel-rich and lithium-rich layered oxide cathodes: progress and perspectives. Adv. Energy Mater..

[12] Yang P (2015). Phosphorus enrichment as a new composition in the solid electrolyte interphase of high-voltage cathodes and its effects on battery cycling. Chem. Mater..

[13] Yan P, Zheng J, Zhang JG, Wang C (2017). Atomic resolution structural and chemical imaging revealing the sequential migration of Ni, Co, and Mn upon the battery cycling of layered cathode. Nano Lett..

[14] Amatucci GG, Tarascon JM, Klein LC (1996). Cobalt dissolution in LiCoO_2_-based non-aqueous rechargeable batteries. Solid State Ion..

[15] Xu K (2014). Electrolytes and interphases in Li-Ion batteries and beyond. Chem. Rev..

[16] Liu H (2017). Intergranular cracking as a major cause of long-term capacity fading of layered cathodes. Nano Lett..

[17] Lee EJ (2014). Development of microstrain in aged lithium transition metal oxides. Nano. Lett..

[18] Kondrakov AO (2017). Anisotropic lattice strain and mechanical degradation of high- and low-nickel NCM cathode materials for Li-ion batteries. J. Phys. Chem. C.

[19] Yan P (2017). Intragranular cracking as a critical barrier for high-voltage usage of layer-structured cathode for lithium-ion batteries. Nat. Commun..

[20] Lim JM (2017). Intrinsic origins of crack generation in Ni-rich LiNi_0.8_Co_0.1_Mn_0.1_O_2_ layered oxide cathode material. Sci. Rep..

[21] Mukhopadhyay A, Sheldon BW (2014). Deformation and stress in electrode materials for Li-ion batteries. Prog. Mater. Sci..

[22] Yin SC, Rho YH, Swainson I, Nazar LF (2006). X-ray/neutron diffraction and electrochemical studies of lithium de/re-intercalation in Li_1–x_Co_1/3_Ni_1/3_Mn_1/3_O_2_ (x=0→1). Chem. Mater..

[23] Ryu HH, Park KJ, Yoon CS, Sun YK (2018). Capacity fading of Ni-rich Li[Ni_x_Co_y_Mn_1–x–y_]O_2_ (0.6≤×≤0.95) cathodes for high-energy-density lithium-ion batteries: bulk or surface degradation?. Chem. Mater..

[24] Sharifi-Asl S (2017). Facet-dependent thermal instability in LiCoO_2_. Nano Lett..

[25] Bak SM (2014). Structural changes and thermal stability of charged LiNi_x_Mn_y_Co_z_O_2_ cathode materials studied by combined in situ time-resolved XRD and mass spectroscopy. ACS Appl. Mater. Interfaces.

[26] Yabuuchi N, Kim YT, Li HH, Shao-Horn Y (2008). Thermal instability of cycled Li_x_Ni_0.5_Mn_0.5_O_2_ electrodes: an in situ synchrotron X-ray powder diffraction study. Chem. Mater..

[27] Lin F (2017). Synchrotron X-ray analytical techniques for studying materials electrochemistry in rechargeable batteries. Chem. Rev..

[28] Karki K (2016). Tuning the activity of oxygen in LiNi_0.8_Co_0.15_Al_0.05_O_2_ battery electrodes. ACS Appl. Mater. Interfaces.

[29] Bak SM (2013). Correlating structural changes and gas evolution during the thermal decomposition of charged Li_x_Ni_0.8_Co_0.15_Al_0.05_O_2_ cathode materials. Chem. Mater..

[30] Qian D, Xu B, Chi M, Meng YS (2014). Uncovering the roles of oxygen vacancies in cation migration in lithium excess layered oxides. Phys. Chem. Chem. Phys..

