# Supplementary material for: Coupling of electrochemically triggered thermal and mechanical effects to aggravate failure in a layered cathode
Source: Nat Commun. 2018 Jun 22;9:2437. doi: 10.1038/s41467-018-04862-w (PMC6014973; doi:10.1038/s41467-018-04862-w)
Supplement: Supplementary file 1 — Supplementary Information [file 41467_2018_4862_MOESM1_ESM.pdf]

Coupling of electrochemically triggered thermal and mechanical effects to aggravate failure in a layered cathode

Yan et al

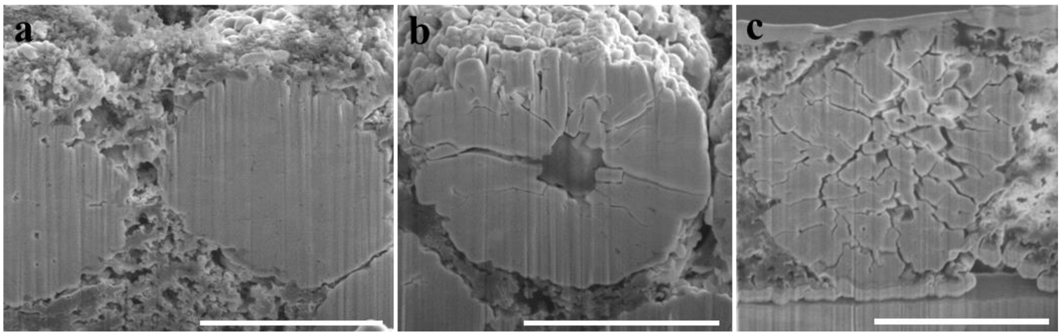

Supplementary Figure 1. Cross sectional images by scanning electron microscopy (SEM). a, pristine sample; **b**, after 100 cycles at 2.7–4.5 V; **c**, after 100 cycles at 2.7–4.8 V. The scale bars are 5 µm.

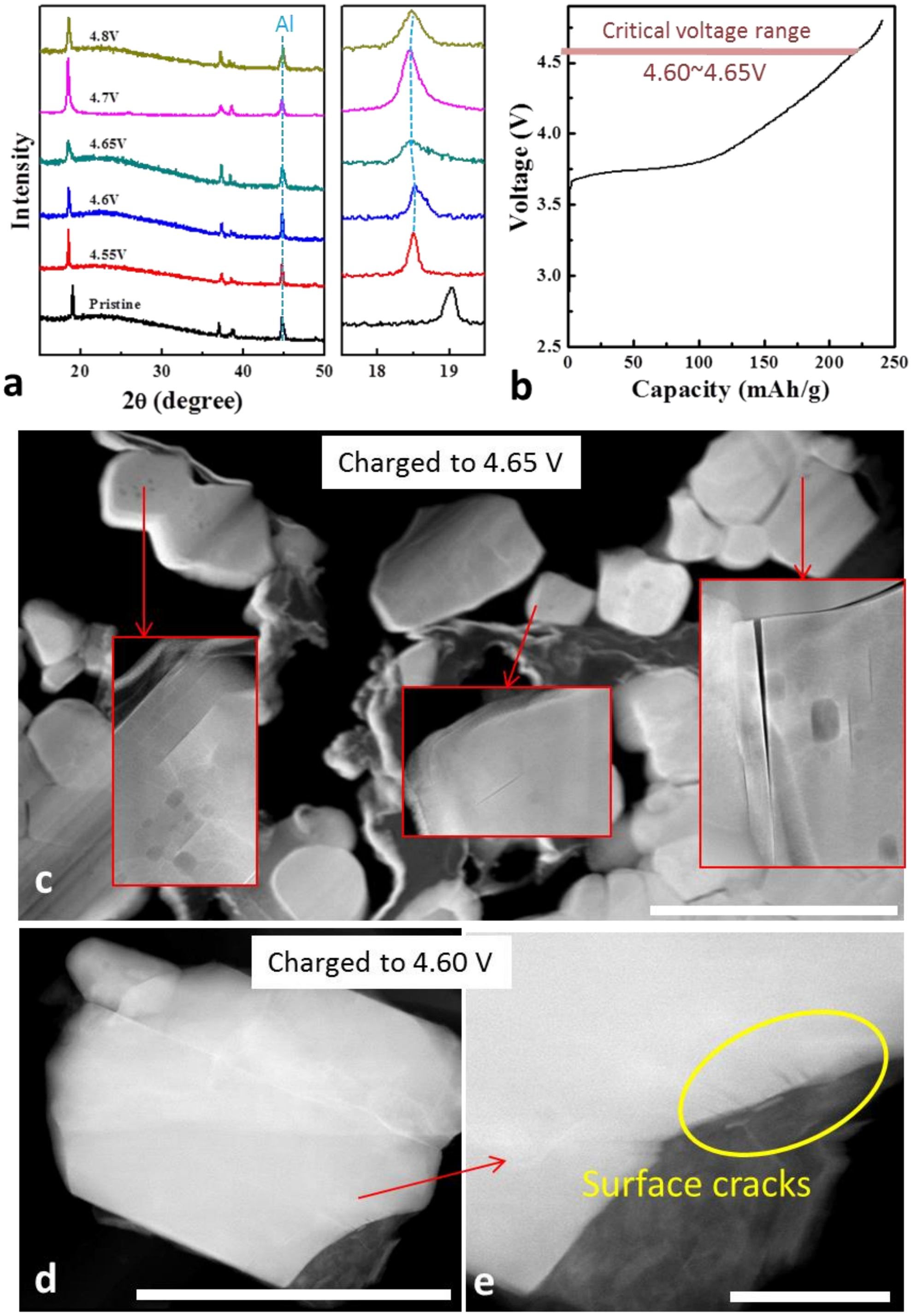

Supplementary Figure 2. Identify the critical onset voltage for intragranular cracks. a, X-ray diffraction (XRD) results of pristine sample and samples charged to 4.55 V, 4.60 V, 4.65 V, 4.70 V and 4.80 V (vs Li metal). **b**, the charge curve and estimated critical voltage range for intragranular cracks. **c**, High angle annular dark field (HAADF) observations on sample charged to 4.65 V. Cracks are shown insets. **d**, Sample charged to 4.60 V does not have intragranular cracks, but it has surface cracks shown in **e** (cycled area). The scale bars are 2 μm in **c**, 1 μm in **d**, and 200 nm in **e**.

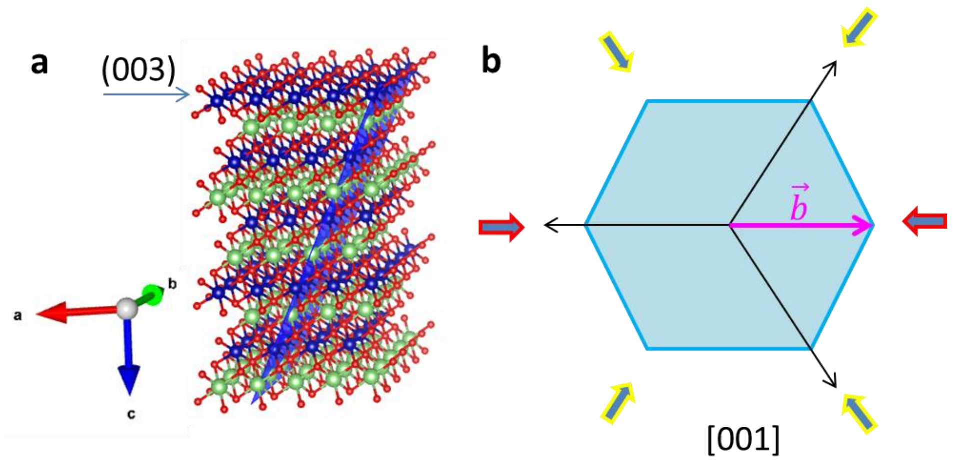

Supplementary Figure 3. Schematically crystallographic analysis. a, The blue plane denotes a (102) plane in layered structure. **b**, Top view from [001] zone axis, red arrows indicate two directions that cannot see extra (102) plane as they are parallel to the Burger's vector $\vec{b}$ Yellow directions can see the extra plane.

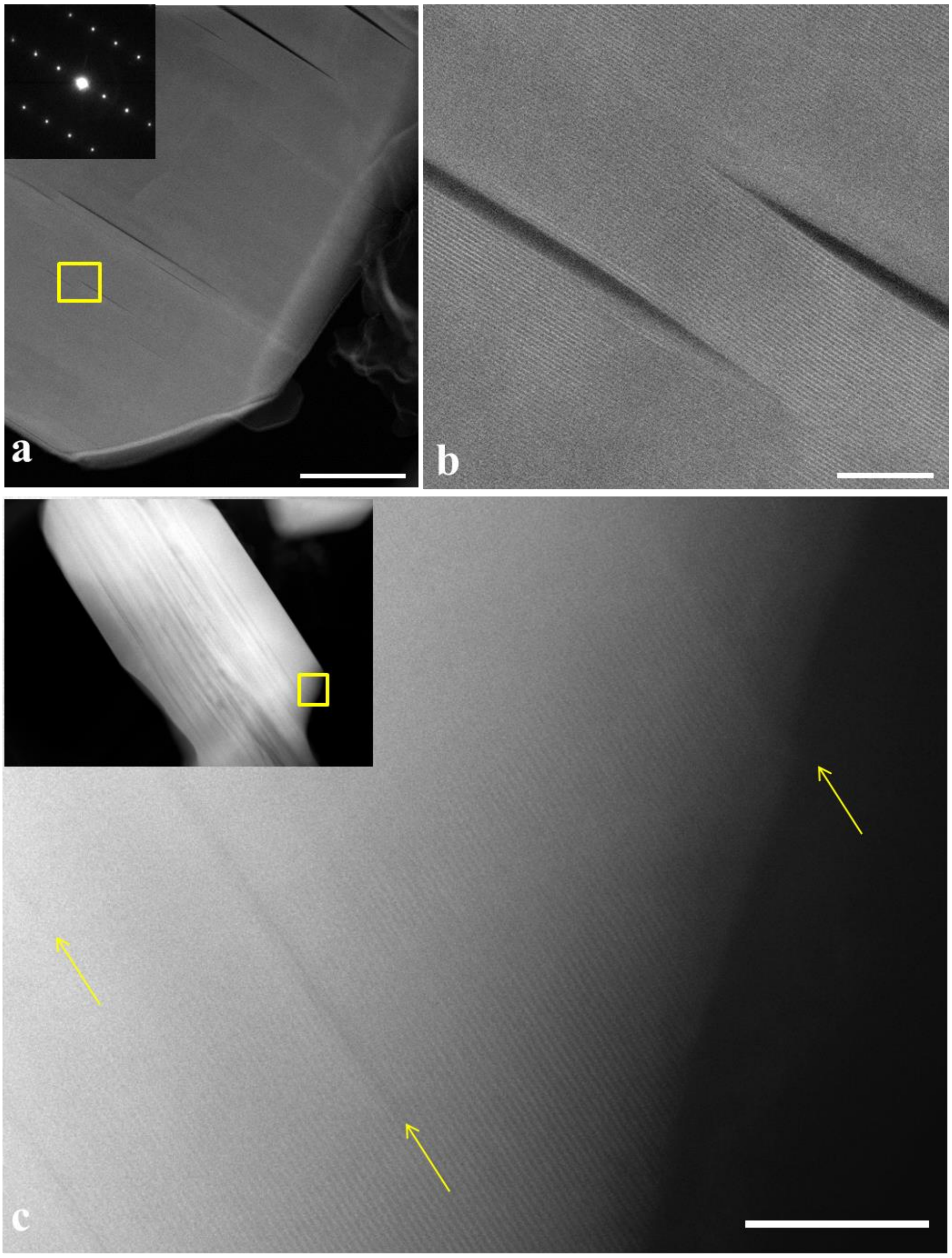

Supplementary Figure 4. Intragranular cracks in delithiated $LiNi_{0.6}Mn_{0.2}Co_{0.2}O_2$. Cross sectional high angle annular dark field (HAADF) images showing real cracks in **a** and **b**, incubation cracks in **c** (yellow arrows). Inset diffraction in **a** confirmes the layered structure. The scale bars are 200 nm in **a**, 10 nm in **b** and **c**.

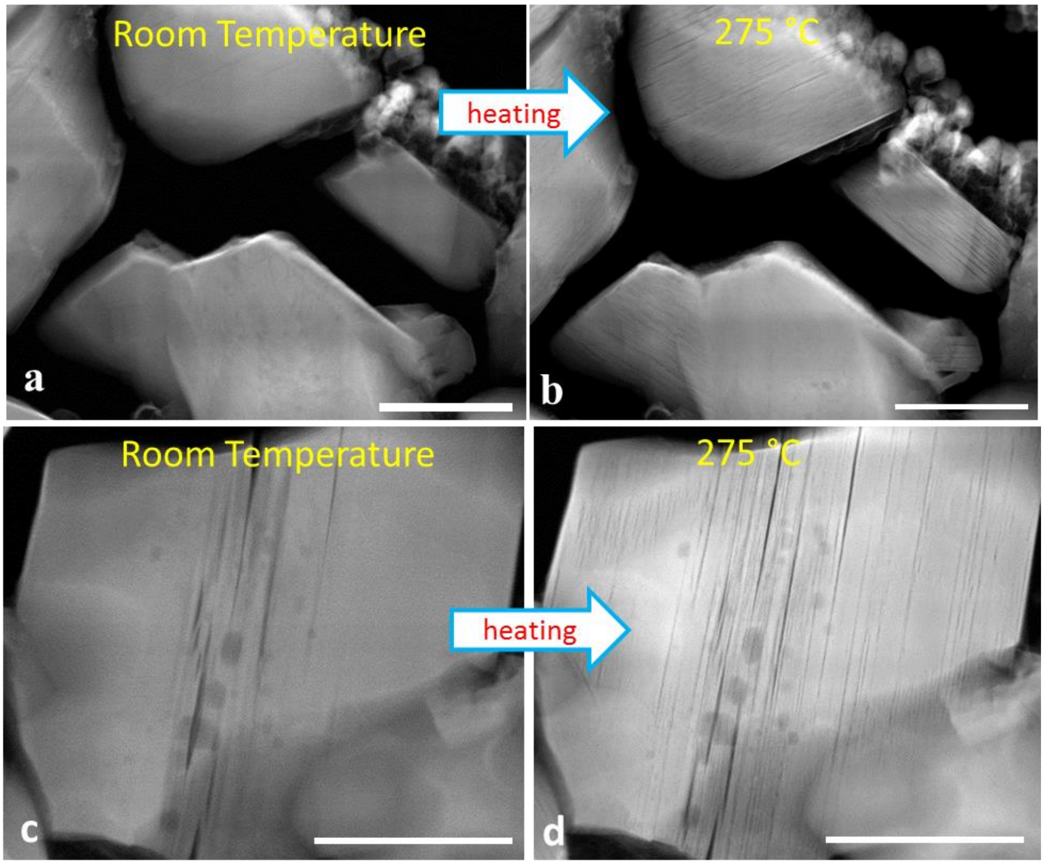

Supplementary Figure 5. In situ heating triggers new cracks. High angle annular dark field (HAADF) images showing cracks formation after heating of delithiated $LiNi_{0.6}Mn_{0.2}Co_{0.2}O_2$ particles from room temperature **a**, **c** to 275 °C **b**, **d**. The Scale bars are 500 nm.

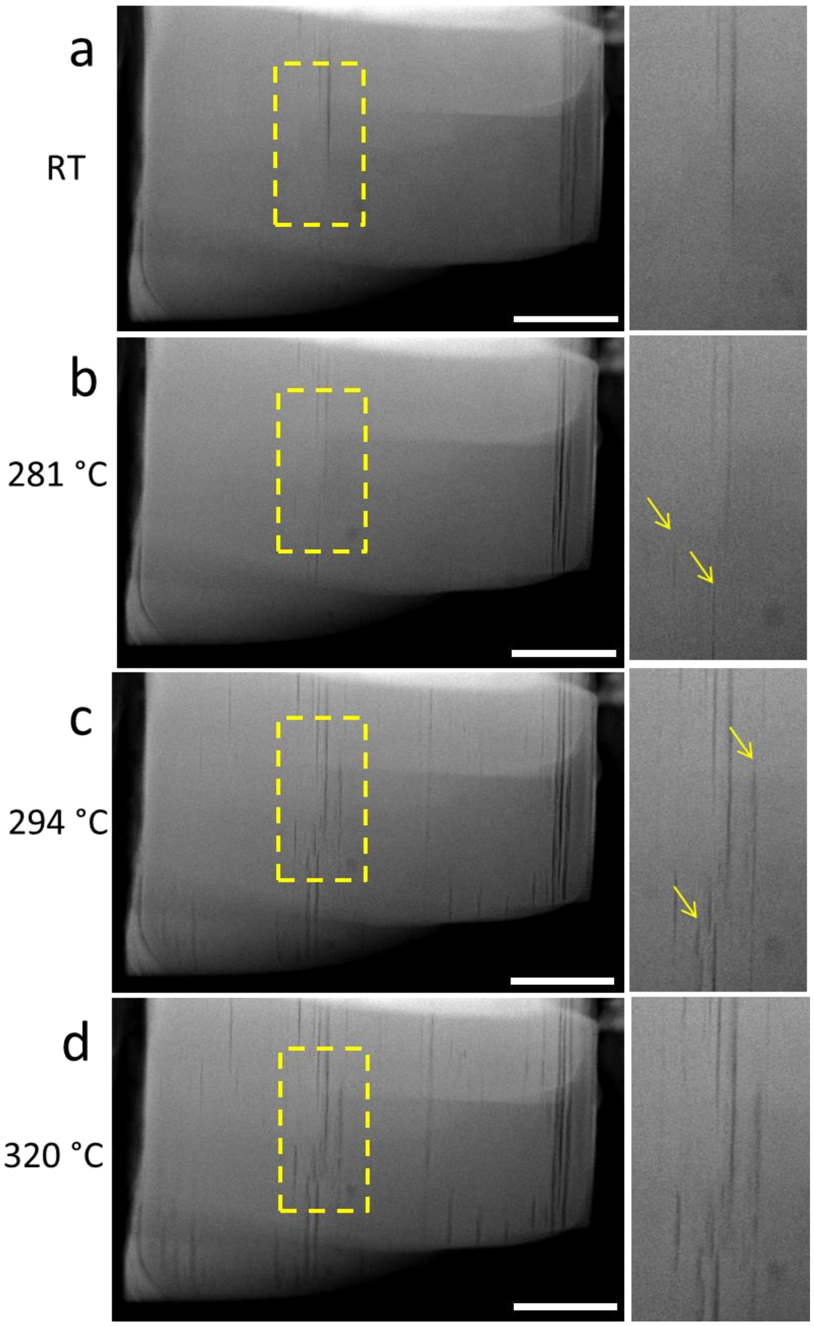

Supplementary Figure 6. Snapshots during in situ heating. A delithiated $LiNi_{0.6}Mn_{0.2}Co_{0.2}O_2$ particle (charged to 4.7 V vs Li metal) is heated from room temperature **a** to 281 °C **b**, 294 °C **c**, and 320 °C **d**. Yellow arrows indicate new cracks. The scale bars are 200 nm.

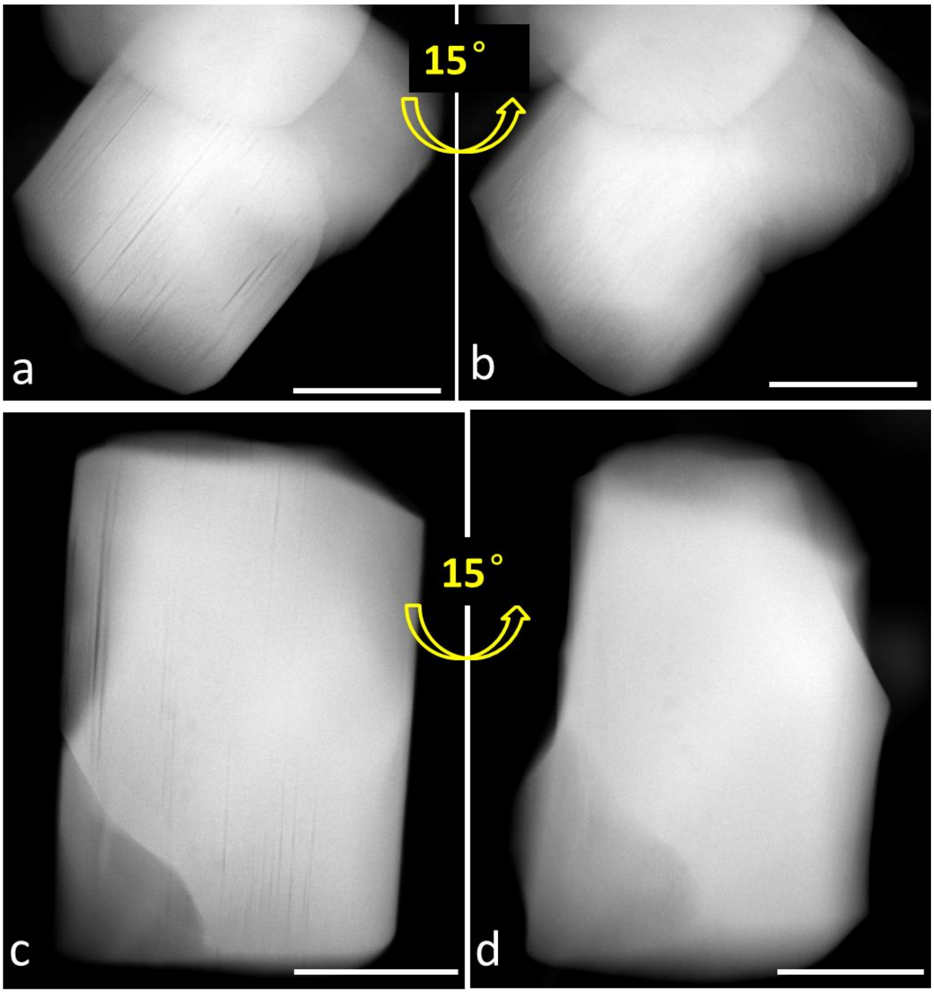

Supplementary Figure 7. Orientation matters crack imaging. Two examples **a**, **b** showing tilting the observation directions can lead to cracks invisible. The scale bars are 200 nm in **a**, **b** and 500 nm in **c**, **d**.

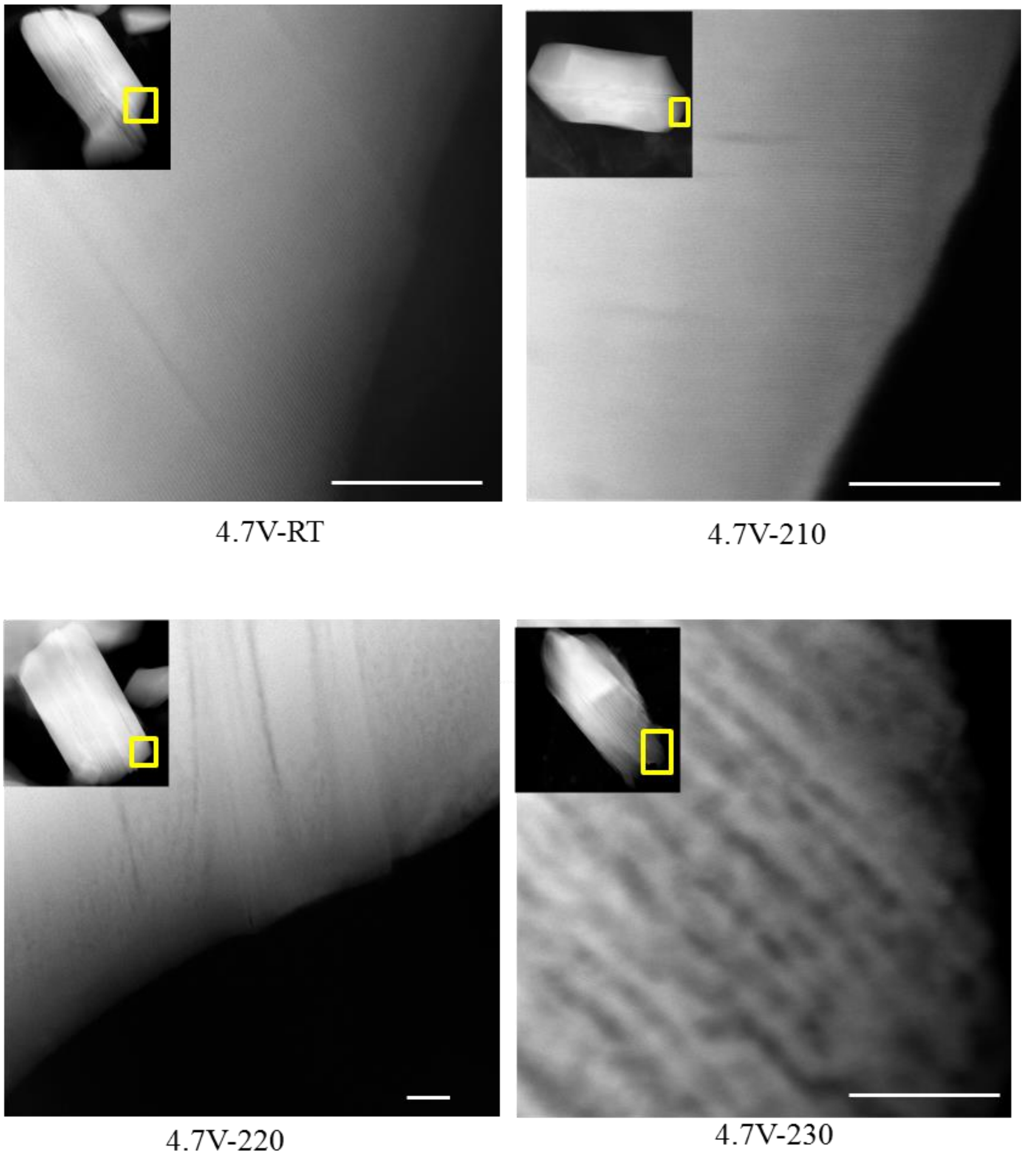

Supplementary Figure 8. Identify the starting temperature point for intragranular cracking and decomposition. High angle annular dark field (HAADF) observations reveal the transition temperature range is 210 °C to 230 °C. The scale bars are 20 nm.

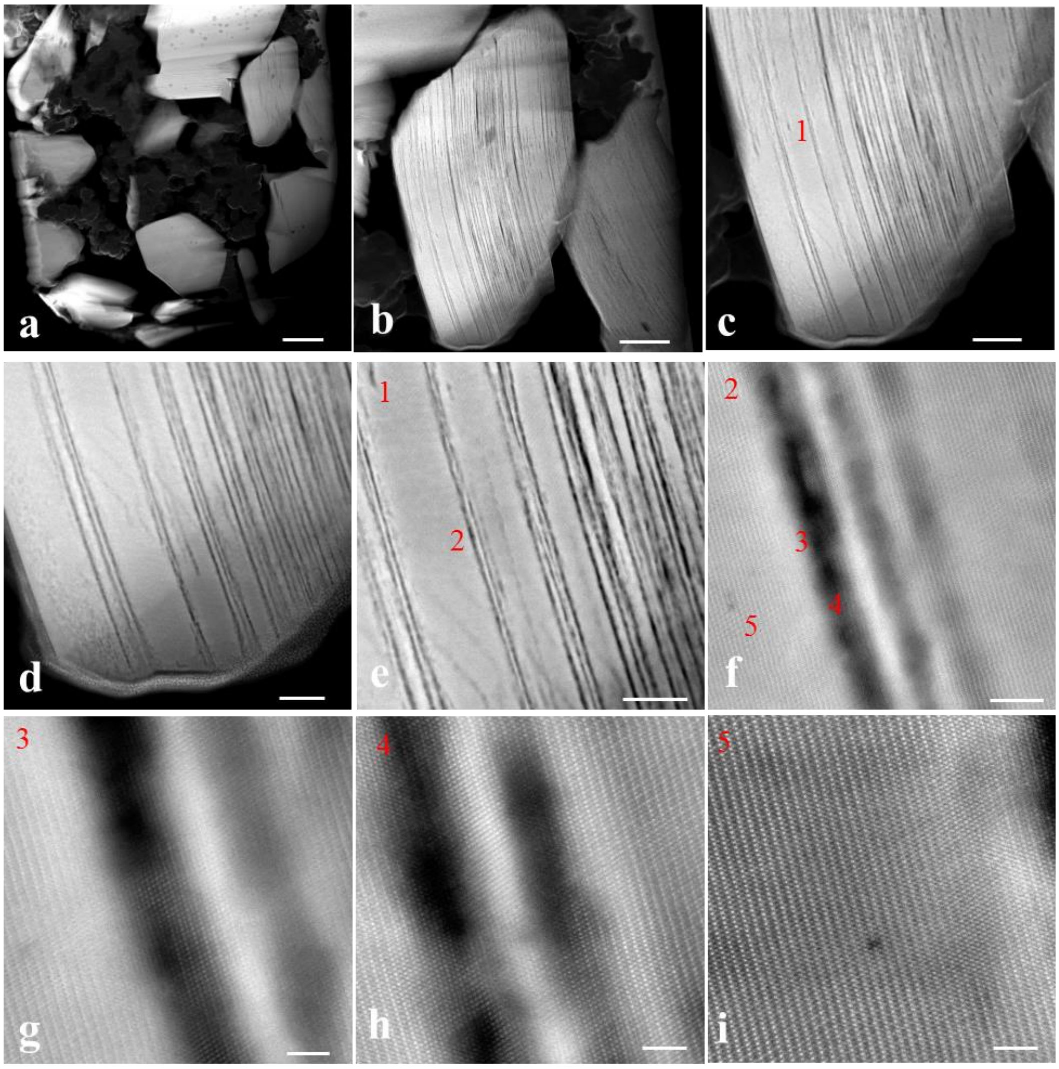

Supplementary Figure 9. Confirming bulk cracking mechanism. Cross sectional observation by high angle annular dark field (HAADF) on FIB milled particles. The sample is heated to 230 °C. Intragranular cracking and thermal decomposition are clearly observed. The scale bars are 500 nm in **a**, 200 nm in **b**, **c**, 50 nm in **d** and **e**, 5 nm in **f**, 2 nm in **g**, **h** and **i.**

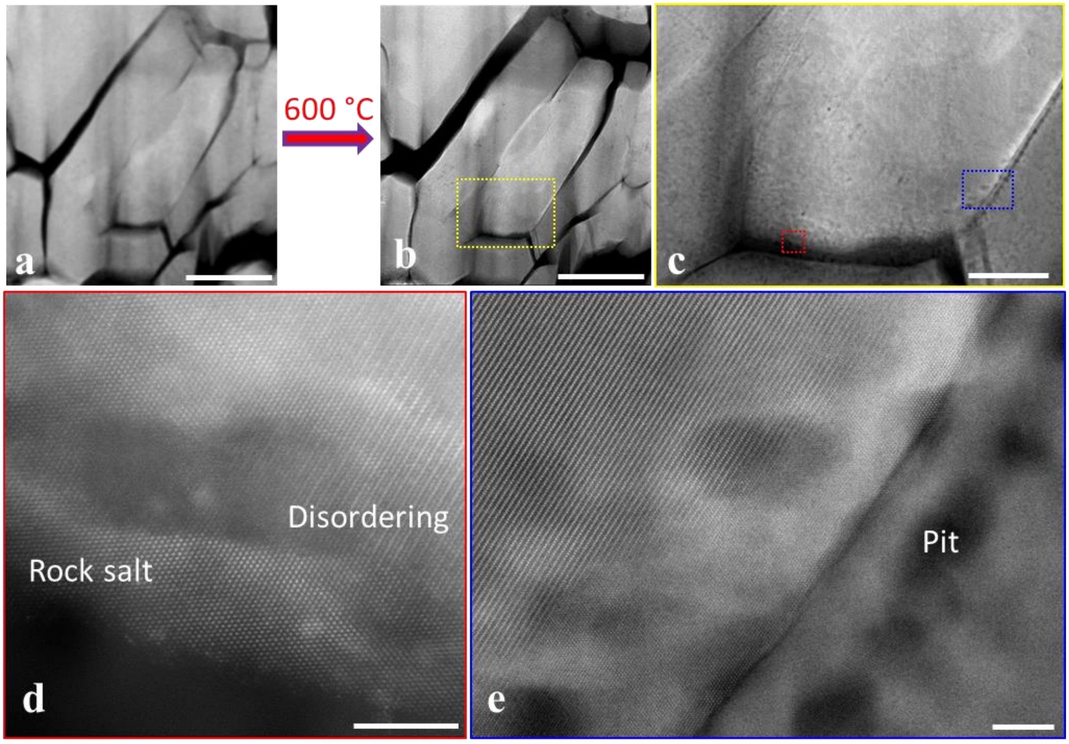

Supplementary Figure 10. In situ heating of fully discharged sample. The $LiNi_{0.6}Mn_{0.2}Co_{0.2}O_2$ sample is cycled 100 times with cycle window as 2.7-4.5 V. **a** the sample area under room temperature, **b**–**e** after heating to 600 °C. **c** is from the yellow frame in **b**. **d** and **e** are from **c**, respectively. Heating resulted phase transformation and pit formation are only located at surface layer. The scale bars are 500 nm in **a** and **b**, 100 nm in **c**, 5 nm in **d** and **e**.

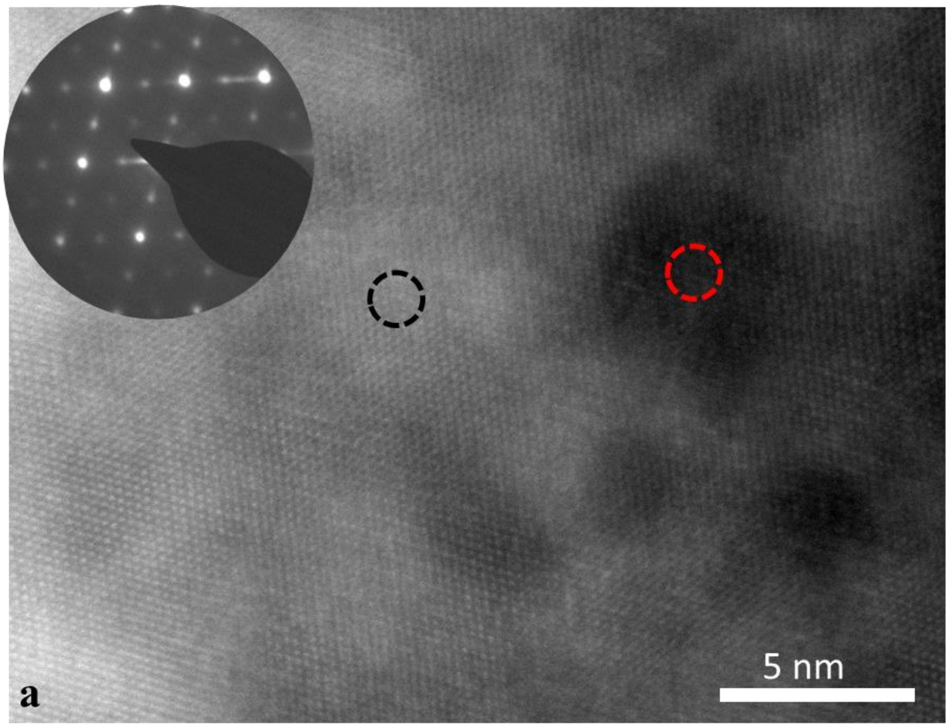

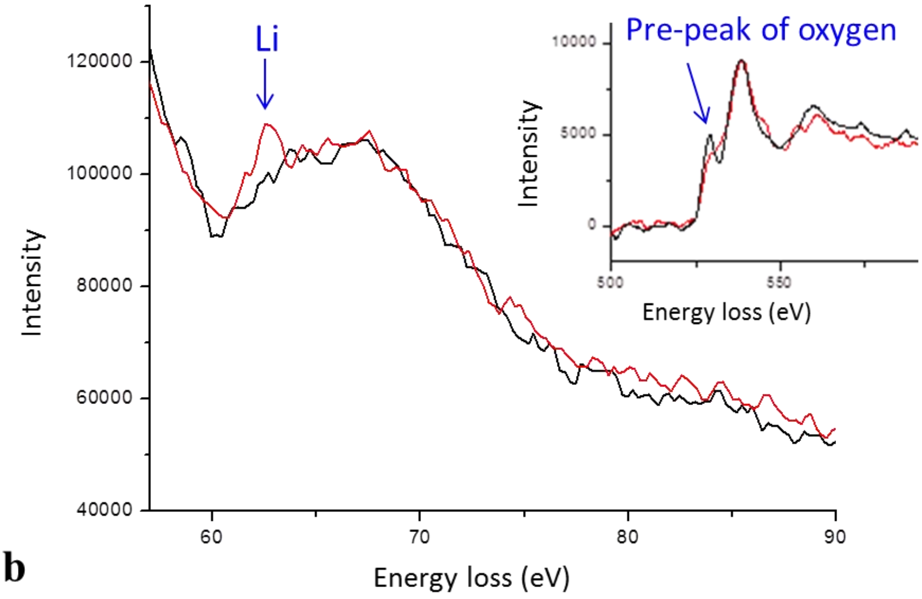

Supplementary Figure 11. **Characterizing pit position. a**, High angle annular dark field (HAADF) image and corresponding selective area electron diffraction (SAED) pattern from thermal decomposed region. HAADF image shows pit formation due to material decomposition. SAED shows a typical spinel featured pattern but without other crystal phase, indicating lithia is in amorphous state rather than crystalline. The scale bar is 5 nm. **b**, Electron energy loss spectroscopy (EELS) measurement from the pit region (red) and original region (black). Pit region shows higher Li concentration evidenced by the boosted Li K-edge. Inset oxygen K-edge also shows a depressed pre-peak which is from lithia.

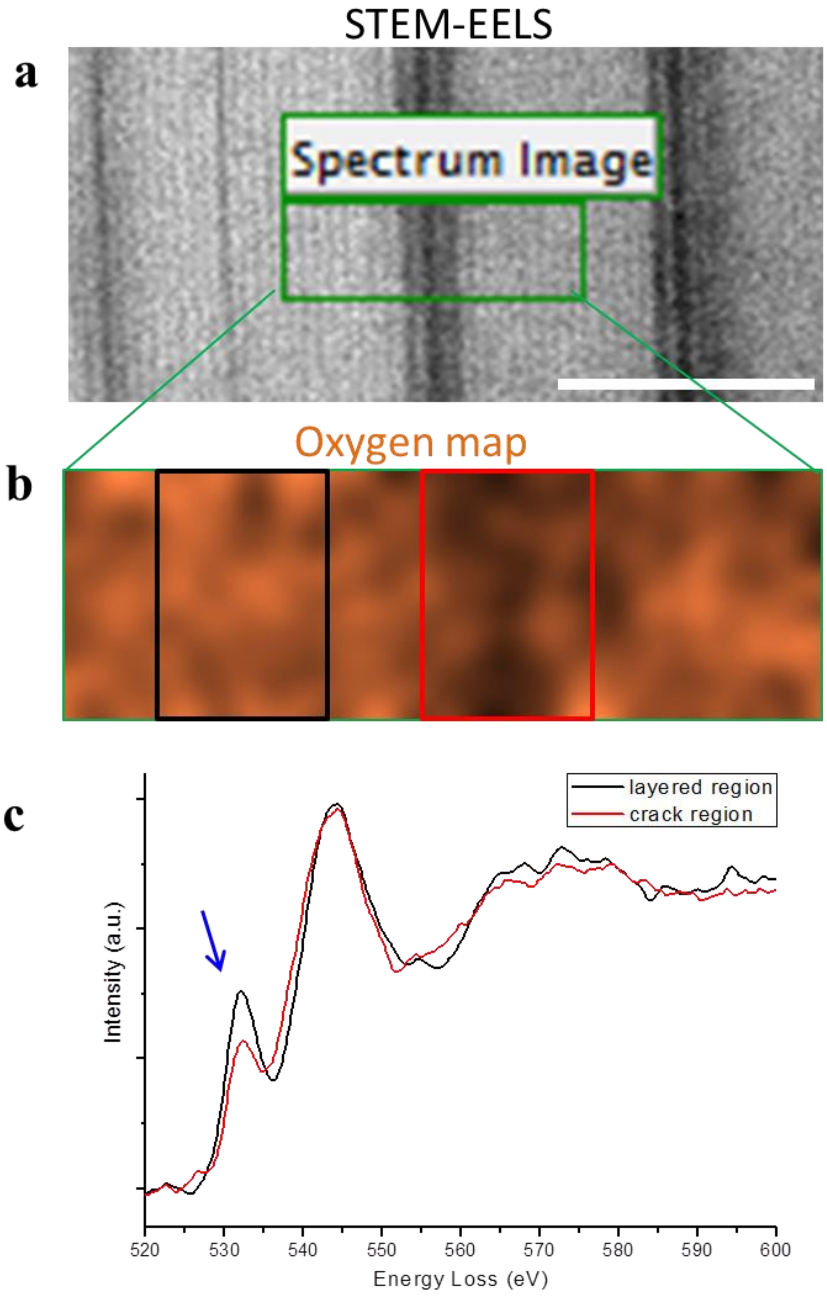

Supplementary Figure 12. Measure oxygen concentration at crack region. a, High angle annular dark field (HAADF) image of cycle induced cracks. The scale bar is 10 nm. **b**, corresponding oxygen map of the region in **a**. **c**, Electron energy loss spectroscopy to compare the crack region (red) and crack-free region(black). The depressed pre-peak of oxygen indicates oxygen vacancies in crack region.

Supplementary Table 1 Mechanical material constants of NMC622 and LiNMC622[3–5]

	NMC622	LiNMC622
E_1	186.7 GPa	229.3 GPa
E_2	186.7 GPa	229.3 GPa
E_3	54.2 GPa	152 GPa
ν_{12}	0.3	0.3
ν_{23}	0.24	0.24
ν_{31}	0.24	0.24
G_{12}	71.5 GPa	95.3 GPa
G_{23}	20.8 GPa	63.2 GPa
G_{31}	20.8 GPa	63.2 GPa

Supplementary Methods

Simulation methods

We developed a mechanics model to elucidate the different origins of the driving forces for crack propagation in the $LiNi_{0.6}Mn_{0.2}Co_{0.2}O_2$ (NMC622) particles. The total strain here is addictive of three components, the chemical (ε_{ij}^{c}), thermal (ε_{ij}^{t}), and elastic (ε_{ij}^{e}) one, as $\varepsilon_{ij} = \varepsilon_{ij}^{c} + \varepsilon_{ij}^{t} + \varepsilon_{ij}^{e}$, where the subscripts $i, j = 1,2,3$ represent the [100], [010], and [001] directions of materials. The chemical strain is induced by lithium insertion, which is assumed to be proportional to the normalized lithium concentration (c) by fully lithiated state, as $\varepsilon_{ij}^{c} = \beta_{ij}c$. The diagonal tensor, β_{ij}, represents the lithiation expansion coefficients. As for NMC622, we set $\beta_{11} = \beta_{22} = 2.8\%$, $\beta_{33} = -4.0\%$, and $\beta_{ij} = 0$ for the other entries.[1] The thermal strain is linearly dependent on the increment of temperature, $\varepsilon_{ij}^{e} = \alpha_{ij}\Delta T$, with the room temperature set as the reference (25 ^{o}C). For layered structured phase (NMC622), we set $\alpha_{11} = \alpha_{22} = 3.34 \times 10^{-6}$ and $\alpha_{33} = 6.3 \times 10^{-6}$ to present anisotropic thermal expansion,[2] whereas the isotropic thermal expansion of RS phase is set as $\alpha = 3.4 \times 10^{-6}$. As an orthotropic crystal, the stiffness tensor of the layered structure depends on nine independent material constants. We set the material constants of NMC622 (c = 0) and LiNMC622 (c = 1) in the model, as shown in **Supplementary Table 1**, and assume that the stiffness tensor of the intermediate stages linearly scales with the lithium concentration (c). However, in an isotropic material like RS phase, the stiffness tensor only depends on two independent constants (i.e. Young's modulus E and Poisson's ratio ν). Here we set $E_{RS} = 180\text{Gpa}$ and $\nu_{RS} = 0.3$. This mechanics model is numerically implemented in the finite element package ABAQUS/standard.

Supplementary References

1. Yoon, W.S., Chung, K.Y., McBreen, J., Yang, X.Q. A comparative study on structural changes of $LiCo_{1/3}Ni_{1/3}Mn_{1/3}O_2$ and $LiNi_{0.8}Co_{0.15}Al_{0.05}O_2$ during first charge using in situ XRD. *Electrochem.Commun.* **8**, 1257–1262 (2006).
2. Hart, F.X., Bates, J.B., Lattice model calculation of the strain energy density and other properties of crystalline $LiCoO_2$. *J. Appl. Phys.* **83**, 7560–7566 (1998).
3. Sun, H., Zhao, K. Electronic Structure and Comparative Properties of $LiNi_xMn_yCo_zO_2$ Cathode Materials. *J. Phys. Chem. C*, **121**, 6002–6010 (2017).
4. Wu, L. and Zhang, J. Ab initio study of anisotropic mechanical properties of $LiCoO_2$ during lithium intercalation and deintercalation process. *J. Appl. Phys.***118**, 225101 (2015).
5. Cheng, E.J., Taylor, N.J., Wolfenstine, J., Sakamoto, J. Elastic properties of lithium cobalt oxide ($LiCoO_2$). *J. Asian Ceramic. Soc.* **5**, 113–117 (2017).
