# Supplementary material for: Coupling of electrochemically triggered thermal and mechanical effects to aggravate failure in a layered cathode
Source: Nat Commun. 2018 Jun 22;9:2437. doi: 10.1038/s41467-018-04862-w (PMC6014973; doi:10.1038/s41467-018-04862-w)
Supplement: Supplementary file 2 — Description of Additional Supplementary Files [file 41467_2018_4862_MOESM2_ESM.pdf]

Description of Additional Supplementary Files

File Name: Supplementary Movie 1
Description: In situ visualizing heating triggered cracking
